# The I's have it: everything needed to practice medical librarianship starts with an I

**DOI:** 10.5195/jmla.2026.2431

**Published:** 2026-01-01

**Authors:** Jean P. Shipman

**Affiliations:** 1 Retired, formerly Vice President, Global Library Relations, Elsevier; and Executive Director, Knowledge Management and Spencer S. Eccles Health Sciences Library, University of Utah, UT

**Keywords:** Janet Doe Lectures, information, professional vocabulary, research, health sciences libraries

## Abstract

The medical or health sciences library professional vocabulary uses many words that start with an *I*. On the eve of the 60th anniversary of the Janet Doe Lectureship, this lecture highlights and summarizes the 15 lectures (27%) that have included an *I* in their titles. The most frequent *I* word was information; this word appeared in four lectures. Only one lecture used more than one *I* word in the title. A new *I* word incorporated in this lecture but not its title is Intelligence, Artificial. +Italics were used to emphasize I words within the lecture or titles of published works.

## INTRODUCTION

Most Janet Doe lecturers have started with some *indication* of the thrill they experienced upon receipt of the notification letter from the Medical Library Association (MLA) stating they were being honored with the Janet Doe Lectureship Award. I too am pleased to be selected to give this *important* lecture. Thank you!

I always joked if I got selected, my lecture would center on how many *I* words exist within our professional vocabulary and jargon. Being the 2025 lecturer gives me the chance to elaborate on this theme.

As this is the eve of the 60^th^ anniversary of the Janet Doe Lectureship, I surmised a review of the Doe lectures featuring an *I* word in their titles would be warranted.

## MARK FUNK

First though, to offer a historical overview of the use of *I* words within our published literature, I consulted Mark Funk's *Journal of the Medical Library Association* paper resulting from his 2012 Janet Doe Lecture entitled “Our words, our story: a textual analysis of articles” published in the *Bulletin of the Medical Library Association/Journal of the Medical Library Association* (*JMLA*) from 1961 to 2010 [1].

His “lecture explored changes in the medical library profession over the last fifty years, as revealed by *individual* word usage in a body of literature” – our association journal.

In Funk's research findings, he noted ‘‘information was the second most used word in the corpus, second only to library.” He also *indicated* “with the *information* world more complicated now, we are doing more teaching, training, and *instructing*.” No surprise, but starting in 1993, the word *Internet* appeared in many journal articles.

## JANET DOE LECTURES

Back to the lectures. To date, there have been 56 Janet Doe lectures given, starting in 1967. Of these 56 lectures, 15 have had at least one word starting with *I* in their titles or 27%.

Before I provide *insights* into the previous use of *I* words within Janet Doe Lectureship titles, here's a short quiz. Match the Janet Doe Lecturer with the corresponding *I* word from their lecture title. As I give my lecture, you can self-correct your quiz. Go.

**Table:** 

a. Louise Darling	1. Idea
b. Betsy Humphreys	2. Implications
c. Julia Sollenberger	3. Inspiring
d. Ana Cleveland	4. International
e. J. Michael Homan	5. Information
f. Sherrilynne Fuller	6. Investing
g. Nina Matheson	7. Index Catalogue
h. Alison Bunting	8. Inside
i. Erika Love	9. Interaction
j. Ursula Poland	10. Intermediary

Now, I will elaborate on each of the 15 Janet Doe lectures containing a title word beginning with an *I*.

The *I* word used most often in Janet Doe Lecture titles is, not surprisingly, *information*. This word appeared in lectures by Jana Bradley, Ana Cleveland, Susan Crawford and Michael Kronenfeld.

### Susan Crawford

When did the *information revolution* or *information society* start? When did it surpass the agricultural, industrial, and service economies? These were the questions addressed by Susan Crawford in her 1983 Janet Doe lecture entitled “The origin and development of a concept: the Information Society” [2].

Crawford's research found the first to discuss the concept of an *information* society was an economist, Fritz Machlup, in 1962, in his book *The Production and Distribution of Knowledge in the United States* [3]. For over thirty years, he *investigated* the production of knowledge and *information* services – a category including libraries and *information* centers. One of his findings was the US “aggregate knowledge production made up 29% of the adjusted gross national product (GNP)…”

In 1969, Peter Drucker continued the discussion with his book *The Age of Discontinuity* [4]. Basing his thoughts on Machlup's, he projected that by 1970, this knowledge sector would comprise 50 percent of the GNP. Crawford claimed this is when the term *knowledge or information society* was coined. The terminology soon appeared in our professional literature.

### Jana Bradley

Jana Bradley offered a different perspective on the word *information*. In her 1995 Janet Doe lecture entitled “The changing face of health information and health information work: a conceptual framework [5], Bradley looked at how our profession was evolving through the lens of outside forces, such as environmental and technological ones, as well as from the viewpoint of other health care professionals – those who could compete for our roles as *information* mutated from print to digital format. She supplied many definitions of *information* and highlighted the many different professionals within health care who handle or manage *information*. She termed a professional as someone with a defined expertise or *identity*. Our profession was changing due to global networked *information* enabled by the *Internet* – making dissemination of *information* easier but also permitting a new composite of *information* of linked multimedia sources and direct connections to other content. The *Internet* allowed *information* to be locally created and published *immediately*. It encouraged simultaneous knowledge generation and publication. Preservation of such *information* was another story, as versioning appeared as a concept and frequent updating possible. What constitutes a document? was a posed lecture question.

The second major change *impacting* our profession at the time *included* the many new approaches to delivering health care. *Institutions* started to place emphasis on assessing their outcomes, competing with others for patients, and implementing *institutional* managed care and practice guidelines, clinical *indicators* and pathways. Many hospitals and centers underwent reorganizations and closures.

Bradley's stance can best be summarized by herself:

Environmental forces such as global networking and changes in health care delivery are changing the cultural facts of health information and the values, practices, and patterns associated with it. Expert information work is changing; new tasks are emerging, and established tasks are changing or diminishing. The temporary balance of roles among the established health professions is being disrupted, and jockeying for jurisdiction will intensify, complicated by overlap of vocabulary, technology, and even some basic tasks. Over time, a new balance of health information professions will emerge, with new tasks, new roles, and new relationships.

Bradley offered *ideas* for how we could assimilate to the changes affecting us to redefine our expertise parameters, as others within the health *information* arena did theirs. She encouraged us to collaborate with other disciplines, but to also maintain and promote our “heartland concepts” and roles to remain vibrant and needed.

### Ana Cleveland

Not surprisingly, Ana Cleveland's 2010 lecture entitled “Miles to go before we sleep: education, technology, and the challenging paradigms in health information” [6] focused on the education of health *information* professionals. As a faculty member of the College of Information, University of North Texas, Cleveland *inspired* us to take action with our education. She felt:

Education for health information professionals must be based on a solid foundation of the changing paradigms and trends in health care and health information as well as technological advances to produce a well-prepared information workforce to meet the demands of health-related environments.

Cleveland believed we could create a new health *information* professional through *intelligent* design and evolution of curricula, framed by an *interdisciplinary* or *interprofessional* group of *instructors* and *individuals*. This meant being trained by those *inside* and outside of our *immediate* field.

Robert Frost's poem “Stopping by Woods on a Snowy Evening” *influenced* Cleveland's lecture title and her thinking that the educational strategy or journey for how future health *information* professionals should be *instructed* could follow four roads.

The first road encouraged us to *identify* what it means to be a health *information* professional. What are our responsibilities, our professional boundaries, and our areas of expertise? The second road emphasized the *importance* of observing changes in our field, as well as those with whom we practice. The third road *insisted* our professional education be based on sound fundamental philosophies. The fourth road was the sharing of Cleveland's *instructional* philosophy. The domain connecting these four roads was *information*.

### Michael Kronenfeld

The 2022 Janet Doe Lecturer, Michael Kronenfeld, challenged us as medical librarians to evolve to assist with the creation, storage, manipulation, and adoption of digital *information* ecosystems, as health *information* professionals. His lecture was titled “2022 Janet Doe Lecture, health science libraries in the emerging digital information era: charting the course” [7]. This transition from object curators to content creators and curators required expanded skills and roles. He credited the National Library of Medicine (NLM), the Network of the National Library of Medicine (NNLM), and MLA for their educational support to enable us to be part of research and clinical teams. These teams assist with describing and managing generated data and evidence for placement into *interoperable* learning storage repositories and tools that guide clinical decisions and data-driven discoveries.

Kronenfeld foresaw an evolution in the development and use of computable biomedical knowledge tools that *integrate* data to analyze and synthesize multiple types and sources of content. These tools guide treatments and personalized medical care. He challenged us to develop *interfaces* to these multiple resources to enable easy access and usability, along with others such as bioinformaticians. A list of perceived new required skills and roles is included in his resulting *JMLA* publication.

### Alison Bunting

Alison Bunting provided an extensive overview of the changes in our profession as reflected in four editions of the *Handbook of Medical Library Practice* and the then forthcoming eight-volume set *Current Practice in Health Sciences Librarianship* in her 1993 Janet Doe lecture (see Table 1). The lecture title was “From *Index Catalogue* to Gopher space: changes in our profession as reflected in the *Handbook* and *CPHSL*” [8].

**Table 1:** Sources Covered in Bunting's 1993 Janet Doe Lecture

1943 – 1st edition	*Handbook of Medical Library Practice*
1956 – 2nd edition	*Handbook of Medical Library Practice*
1970 – 3rd edition	*Handbook of Medical Library Practice*
1982 – 1988 4th ed.	*Handbook of Medical Library Practice*
1996 – 2000	*Current Practice in Health Sciences Librarianship, 8 vols.*

My personal reflection upon reading Bunting's lecture is – my, how times have changed. In the fifty years covered by her lecture (1943–1993), there was a recognized ability to standardize many medical library practices and to define key areas of responsibility. I venture to say, post the digital transformation of tools and content, librarians have tended to differentiate their practices to fit their local *institution's* strategic directions. The library started to become the center for campus activities and *initiatives*. In addition, many of the key roles outlined in Bunting's lecture have been largely assumed by library technicians or paraprofessionals. Roles remaining constant – but assuming a greater *intensity* over time – include *instruction* and service.

But what happened as far as practice transformations in the 50 years *investigated* by Bunting? I’ll recap.

The overarching change over the years of the *Handbook* was the acceptance of the medical librarian as being the one to administer the library versus physicians. As the size of libraries grew, library directors became more *involved* with administrating libraries, and then they became more outwardly focused – with the technical work being completed by other hired librarians.

Collection selection progressed from the review of published bibliographies by professional organizations and large library acquisition lists to vendor support and approval plans. Over time, books became less important and journals more so. Cataloging changed. Instead of selecting subject headings from entries in the *Index Catalogue*, Medical Subject Headings (MeSH) and the NLM classification system offered authoritative vocabularies.

The amount of *Handbook* content dedicated to library administration *increased* over time. Personnel management *issues* first appeared in the fourth edition, including topics such as recruiting, *interviewing*, hiring, and assessing employee performance.

NLM was not given its own chapter until the third edition of the *Handbook*. The fourth edition included even more coverage of the Regional Medical Library (RML) network and *interlibrary* cooperation.

Reference services and *instruction* were covered in every edition. Focus shifted from print reference resources to the development of policies and discussions of *interview* styles, to how access to *information* was facilitated by electronic databases and how to conduct mediated searches.

The last areas of comparison in Bunting's lecture dealt with emerging technologies, *including* their *impact* on the sharing of journal articles through *interlibrary loan* and document delivery, and the tools used to deliver content, such as fax machines and photocopiers. Resource sharing tools, such as DOCLINE and the OCLC *interlibrary loan* system, appeared in the fourth edition of the *Handbook*.

### Scott Adams

Ok, how many of you have done a PubMed search? How many of you have ever thought about MEDLARS's (MEDLINE) origin? I honestly have to say my energies were focused on how to effectively search this database of bibliographic citations and abstracts, and I never really thought about who or what occurred to *implement* this ubiquitous system.

To learn about the history of the creation of MEDLARS, I recommend reading Scott Adams's Janet Doe lecture given in 1972 “The way of the innovator: notes toward a prehistory of MEDLARS” [9] – over 50 plus years ago.

Several take aways for me from Adam's lecture include: 1) this was a great example of *industry*, government, and association collaboration, 2) it *involved* dedicated *individuals interested* in *indexing* and description to design the database *infrastructure*, 3) MEDLARS took over 15 years to conceive prior to its contractual development with *industry*, and 4) the *influence* Janet Doe and other MLA luminaries had with its *innovation*.

Janet Doe, you say? Yes, she served on three formative committees and Adams credits her for “the concept of publishing multiple specialized indexes from a common data base, which came to fruition in the MEDLARS recurring bibliographies...”

A partnership with the academy and government was achieved through a contract with the Army Research and Development Board and the Johns Hopkins University. This 1948 contract created the Welch Medical Indexing Project with this charge (see Table 2).

**Table 2:** 1948 Welch Medical Indexing Project Charge

To explore the volume of medical literature To determine the coverage of this literature by existing bibliographic resources To note the commonalities and differences of subject descriptors among these existing resources, and To determine if indexing could be automated

After receiving development funding of $500,000 from the National Heart *Institute*, NLM solicited proposals in early 1961 from *industry*, based on the final technical specifications. General Electric Corporation won the MEDLARS development contract, and MEDLARS was released three years later, in 1964, costing a total of $3 million.

Having worked directly with *innovators* at the University of Utah, I understand how difficult collaborations between different types of agencies can be and how long a product can take from *ideation* to *implementation*. MEDLARS proved to be no different. *Innovations* of this magnitude take years, but we can see how *influential* the MEDLARS development pioneers were to our profession. Their visions and efforts have survived the test of time and continue to serve health care well.

### J. Michael Homan

It's a pleasure to *inform* you that J. Michael Homan wins the prize (pun intended) for using two or three *I* words (depending on how you want to “look” at it) with his 2009 Janet Doe lecture titled “Eyes on the prize: reflections on the impact of the evolving digital ecology on the librarian as expert intermediary and knowledge coach, 1969–2009” [10]

Homan believed medical librarians could efficiently and effectively contribute to the success of *individuals,* and *impact* their *institutions,* with their *intermediary* expert literature retrieval skills and ability to synthesis the literature. These roles resulted in time savings for other *institutional* health care experts, as they could apply supplied *information* to their decision making. The role also ensured a place at the table for librarians within committees and teams conducting research, patient care, and *instruction*. Homan provided evidence for his stance from his forty-year career.

Homan directly observed the *informationist* role – called an embedded analyst – at the Upjohn pharmaceutical company before the *informationist* word was coined by Davidoff and Florence in 2000 [11]. The request for a librarian to be a part of a drug development team was *initiated* by a library user.

Little did Homan know that the *informationist* concept would be a key topic of his MLA presidency. He appointed a task force to plan an NLM sponsored conference to explore the topic in 2002. I recall reading the MLA Board meeting preparatory documents to discover that I was going to be the chair of this task force. I walked the streets of Richmond, Virginia, in a daze thinking how in the world was I going to accomplish this task. With the help of many, the conference was a success.

Homan was one of the first MEDLINE trainers when he was employed by the University of California at Los Angeles (UCLA). The UCLA Biomedical Library served contractually as one of several MEDLARS search centers across the country and world. Users could submit their questions to a search center and a batch literature search would be conducted by NLM within a three-week time period. A printout of results would be mailed to the search center to be given to the requesting user. Training of staff for a search center took three weeks at NLM. Louise Darling, director of the UCLA Biomedical Library, felt that a training center should be established on the West coast to enable more librarians to obtain the necessary training. Funding for such a training center at UCLA was achieved through an RML contract.

We were worth our *institutional investment* in Homan's eyes, and he encouraged us to keep our eyes on this value as the digital ecology around us changed. He stated “Our experienced knowledge coaches are the marriage of librarian expertise and high-tech and soft touch personalized service. It will always be a winning combination.”

### Betsy Humphreys

In Betsy Humphreys’ 2001 Janet Doe Lecture, she included the word *interactions* in her lecture title “Adjusting to progress: interactions between the National Library of Medicine and health sciences librarians, 1961-2001” [12].

This lecture reviewed two major changes to NLM's mission over a forty-year period affecting the relationship and *interactions* between NLM and health sciences librarians over a forty-year period. These two major changes included the *implementation* of the National Network of Libraries of Medicine (now the Network of the National Library of Medicine) and direct service outreach by NLM to *individual* health care providers.

The resulting JMLA publication abstract from Humphreys’ lecture includes four *I* words – *implementation, individual, intermittent,* and *irritation*. The last *I* word, *irritation*, was the result often felt by librarians when NLM offered new, changed, or deleted services. Luckily, the *intermittent* word reflected that relationship woes between NLM and librarians were often short-lived and *issue*-specific. In fact, many past Janet Doe lecturers included sections within their talks about the relationship between MLA and NLM and about NLM's positive *influence* on our profession.

As I traveled across the country and globe when I was with the different RMLs, Elsevier, and also as MLA president, I learned of the jealousy existing among academic librarians for our deep connection to, *interaction* with, and dependency on the NLM. No such entity exists for academic librarians. NLM has enabled us to conduct our responsibilities with relevant technologies and has employed *individuals* who envision and create tools and knowledge to support our collective professional needs.

### Erika Love

One of the many benefits of giving the Janet Doe lecture is taking the opportunity all of us have, but many of us don’t accept, to read past Doe lectures. Of those I have read, there is one *individual* whom I regret never having met. I feel a bond to this *individual*, as I agreed with so many of her visionary comments. The difference is I agreed with many of her visions after they became reality. To have been able to perceive the future the way this librarian did is mind boggling to me. This person is Erika Love, past library director, Medical Center Library, University of New Mexico. Love's 1987 lecture was entitled “The science of medical librarianship: investing in the future” [13].

Most of Love's 1987 lecture focused on what medical librarianship and libraries should be in the 21^st^ century. She wondered could we as a profession survive? Her fear was not based on libraries becoming extinct, even though she shared thoughts of others who felt so. No, her fear was we would not do enough *investing* in research to maintain a unique professional knowledge base, as others vied for the *information* arena.

She acknowledged several transitions *impacting* our future. First, the change in the type of work librarians performed – it became more managerial and *instructional* than clerical. The transfer of clerical duties to technical staff she felt warranted a review of our *identify* as librarians. She encouraged us to offer our technical staff more skill development opportunities and recognition. She also felt the name for technical staff should be standardized and research on training costs for them should be performed.

Second, there was a lot of discussion about who would be the *information* managers of the future – librarians or commercial employees. Love perceived a role for both, but felt librarians would be more *impartial*, as they would not be profit-driven. Librarians would be concerned about the quality of *information* and its preservation over time – ensuring the voices of many diverse *individuals* would be recorded. She was worried about deemphasizing the ownership of materials and expressed concern over who controlled the gateway to access to *information* in an electronic world.

Third, Love's vision of the library of the future was one that would collect fewer physical materials but exerted some control over access to digital *information*. Libraries would serve as a *social institution* where people gather to share *ideas*.

As the director of the health sciences library at the University of Utah, I often was asked “What is today's library?” especially since we had discarded most of our print collection to create space for a medical gaming lab and a center for *innovation*. My response was – We are in the business of collecting people; our physical space serves as a study space, a meeting center, and an i*deation* and prototyping space. Our *information*al content, once contained in physical books and journals, is mostly digital – permitting accessibility from office, home, or lab. Librarians are liberated and can engage with you within your context. We still provide *information* through what we license and make discoverable; but in freeing ourselves from a print collection, we are able to offer more *instruction*, outreach, and create digital educational and repository resources. In other words, we assist with knowledge creation as a peer.

I could go on about Love's lecture, but I will stop and suggest you *invest* time to read it. I will share a few favorite words I found within the lecture: *information* empires (libraries), *intellectual* leadership, geographical *immobility* (library handicap), technological *imperative*, and *intellectual* dependence (if we don’t do research).

### Sherrilynne Fuller

“Enabling, empowering, inspiring: research and mentorship through the years” [14] was the 1999 Janet Doe lecture given by Sherrilynne Fuller. I recall enjoying this lecture at the 99^th^ annual meeting of MLA, but I also really enjoyed reading it as I prepared for this lecture.

I was employed by Fuller at the time at the University of Washington Health Sciences Library and Information Center in Seattle, Washington. I found Fuller's energy, passion, and *intelligence invigorating* and *indeed inspiring*. Through the efforts of Fuller, I learned how to license content as free-form *information* – not packaged within containers, such as journals or books. She was mining data before it was cool! I also witnessed her desire and ability to collaborate with others throughout the *institution*, not only with other librarians. She *illustrated* how *information* could permeate a campus and be accessed and applied outside library walls.

Via her lecture, Fuller challenged us to be researchers and practitioners; research was not just for “ivory tower academics.” She shared how this concept was not really a new one, but one still needing to grab some traction and *implementation*. In fact, she believed our ability to *isolate* an *issue* of *interest* and conduct a scientific and *impartial investigation* of the *issue* was paramount to our profession's survival. We needed to demonstrate our value through conducting research and mentoring others to do the same. In her viewpoint, research and mentoring should be *interwoven* and equally *important*. Collaborative research by *individuals* within and across *institutions* was enthusiastically supported – team science as we term it today.

### Nina Matheson

The 1994 Janet Doe Lecturer was Nina Matheson, who talked about “The idea of the library in the twenty-first century” [15]. Matheson shared her two main professional career *ideas*. “One idea is that librarians and libraries must be agents of change.” She referenced her famous *IAIMS* model – the *Integrated* Advanced *Information* Management Systems – published in 1982 [16]. “The other idea is that the fundamental idea of the library must change, that our business should be the ownership and management of first-hand knowledge rather than the mere storage and dissemination of second-hand knowledge.”

Unlike Fuller, Matheson was not convinced we should conduct research on operational library matters. While this was useful in an “*industrial* capitalism” world, it would not be viable in a “knowledge capitalism” era. “Knowledge in the next era is a capital resource. The talent and ability to apply knowledge to create knowledge and to organize it for useful purposes will be fundamental to the survival and growth of organizations as well as individuals.”

Matheson looked to new *information* technologies such as Mosiac, the World Wide Web, and the *Internet* as game changers, as they offered the capabilities of linking different forms of *information* from around the globe to produce knowledge. She championed librarians to work collectively to not replicate digital libraries at the *institutional* level, but to think of what we could create to be shared. An example she offered was the Genome Data Base (GDB) – the effort to map the human genome. This human map could be federated with other living species genetic maps to formulate an *Encyclopedia of Life*. This to her would be a 21^st^ century library – a viable, ever-changing database or knowledge base of first-hand *information* – a knowledge server. This is the library she envisioned for the future.

I worked with Matheson and could see the visionary she was up close and personal. She foresaw a different skill set for librarians, as she believed we could help to create and manage knowledge. The *IAIMS* model placed the library as central to campus – not physically, but philosophically. She encouraged health care professionals to consider *information* and knowledge as central to their work and advancement.

### Ursula Poland

Many of you have heard of the Cunningham Memorial International Fellowship, but do you know of its origin? In her 1982 Janet Doe lecture “Reflections on the Medical Library Association's international activities” [17], Ursula Poland provided a historic overview of MLA's *involvement* with *international* libraries and cooperative programming. This topic was deemed appropriate as Janet Doe served as the first chair of MLA's Committee on International and National Cooperation formed in 1948. Doe was appointed by the MLA president, Eileen Roach Cunningham. Cunningham worked on MLA's behalf on *international* efforts with UNESCO and with a key *initiative* to train medical librarians from other countries. Through her estate, Cunningham left funding for such a program in 1971, commonly known as the Cunningham Fellowship. Funds were provided to an *international* librarian selected by the International Cooperation Committee to travel to the US to spend time with a library and its staff to learn about medical librarianship, often with the requirement of the fellow presenting about one's experience at the annual MLA meeting. This program continues today.

The MLA committee dealing with *international issues* underwent several name changes from its beginning (see Table 3).

**Table 3:** Progression of International MLA Groups

1948 – 1950	Committee on International and National Cooperation
1950 – 1976	Committee on International Cooperation
1976 – 2019	International Cooperation Committee
2019 –	International Cooperation Caucus

A summary of activities of the existing International Cooperative Committee concluded Poland's lecture. This summary was accompanied by her plea to MLA to continue to be *involved* globally. She encouraged *individual* members to join other national library associations to learn of their *issues* and events and suggested medical librarians consider personnel exchange programs among countries.

### Martha Jane K. Zachert

An *inquiry* into our professional values was conducted for the 1978 Janet Doe lecture given by Mary Jane K. Zachert. Her lecture title was “Books and other endangered species: an inquiry into the values of medical librarianship” [18].

To *identify* our shared values, Zachert reviewed 28 plus volumes of two of our field's journals and past Janet Doe lectures (see Table 4). She admitted however to letting her knowledge of the field, her *insights* from attending conferences and *interacting* with medical librarians, and her reviews of MLA actions as *impacting* her conclusions as well.

**Table 4:** Sources Reviewed for Zachert Janet Doe Lecture

*Medical Library and Historical Journal*, 1903–1907
*Bulletin of the Medical Library Association*, every 4^th^ year between 1911 and 1977
Janet Doe lectures

I’ll summarize her findings; however, I do recommend a read of her lecture to glean all of the nuances. The most predominant value she discovered was “professionalism.” Others included “cooperation, a sense of community with health sciences practitioners, and knowledge orientation.”

Cooperation occurred more among ourselves and not so much as partnerships or collaborations with others within our *institutions*. In early MLA years, many doctors were members and leaders of MLA. Later, health care providers became more our audience – or those to whom we offered resources and services. *Knowledge orientation* dealt with the *idea* we curated the health sciences’ knowledge base by describing it, acquiring it, organizing it, storing it, and delivering it. In addition to providing a “keeping*”* function, she suggested we create knowledge by applying the scientific method to conduct our own research.

The profession started to explore certification as a means of creating some organization about what we do. Certification was one attempt to *identify* qualified professional librarians. At the time, MLA offered a certification examination, a precursor to the Academy of Health Information Professionals. Certification also encouraged education post the formal degree – aka continuing education.

Zachert ended her lecture by posing many questions and encouraging us to *inquire* about the answers. Many dealt with our self-*image* as a profession – what is our expertise and how do we differ from other librarians, if we differ? Do we need more rigorous scientific research performed about our values so we can indeed confirm them, commit to them, prioritize them, and deal with changing them over time, as warranted?

### Louise Darling

Most of us have heard of AHIP or the Academy of Health Information Professionals (AHIP) which was *instituted* in 1988. But how many of us know the history of MLA's certification programs over the years and its *implications*? I was *intrigued* by the history given via Louise Darling's 1979 Janet Doe lecture entitled “The view behind and ahead: implications of certification” [19].

This lecture was given during MLA's 75^th^ anniversary, 25 years into the MLA Certification program. It was dedicated to Janet Doe, as she was a major proponent for MLA to have some formal qualification recognition program. In fact, the Code for the Training and Certification of Medical Librarians was adopted during her presidency, at the 1949 annual MLA conference. This was the first professional association attempt at establishing criteria and competencies for medical librarians.

A walk down memory lane of how medical libraries came to be was fascinating and covered why there had not been a training or certification code to date. It also explained why such a code *initiated* controversy and concern among our profession.

MLA started its certification program with a three-tiered system governed by the Committee on Standards for Medical Librarianship in 1949. There was not wide consensus about the value of the program, and there was a lack of member *interest* to provide feedback about what should be *included* in such a program. Darling provided several reasons for this. She felt certification needed to be given due attention, as the number of health care facilities and medical libraries *increased*, along with the volume of health *information* published. She recommended a “fairly simple new code that will require a minimum amount of interpretation” be considered.

### Julia Sollenberger

In 2017, Julia Sollenberger encouraged us to look *inside* ourselves with her Janet Doe lecture entitled “Looking inside ourselves: a culture of kindness [20]. *Inspired* by programs offered at the University of Rochester Medical Center, where she directed the library, Sollenberger reminded us if we take care of ourselves, we are much more likely to *improve* our *interpersonal* skills and to express kindness to others in our *interactions*.

Sollenberger reflected on a personal mindfulness training course, which made her more aware of her surroundings, others in the program, and of her own actions and thoughts. Many companies and large health care centers offer programs like the one she attended to encourage their employees and health care providers to communicate and listen *intently* to patients and clients with whom they *interact.* A key part of looking *inside* oneself *included* examining one's emotional and social *intelligence*.

I found numerous other words within Sollenberger's lecture starting with an *I* including: *integrity, isolated, illness, incident, impression, inadequacy, initiatives, invitation, intrigued, information, instructions, interpretations, innovative, insight, intensity*, and buttermilk *iced* cookies. Now you have to be *intrigued* by that last item!

## QUIZ ANSWERS

It's time to assess your learning. Here are the answers to the earlier quiz. Did anyone get them all correct?

**Table:** 

a. Louise Darling	1. Implications
b. Betsy Humphreys	2. Interactions
c. Julia Sollenberger	3. Inside
d. Ana Cleveland	4. Information
e. J. Michael Homan	5. Intermediary
f. Sherrilynne Fuller	6. Inspiring
g. Nina Matheson	7. Idea
h. Alison Bunting	8. *Index Catalogue*
i. Erika Love	9. Investing
j. Ursula Poland	10. International

Let's pause for a slight *intermission* as I transition from the historical part of my lecture to the philosophical portion. Take a minute to reflect upon the *I* words impacting you the most personally, and for the profession.

What *I* words do you think will comprise our future? What *image* do I wish to portray? What differences can *I* make as a medical or health sciences librarian? What is in our collective crystal ball?

As I peer into the future, I see *innovation, ideation,* and *imagination* still being major needs and characteristics of our profession. We will continue to adjust to changes in technology, curricula, research methodologies, and clinical care. I also see *intelligence* as being key, especially *artificial intelligence or AI.* AI will create a revolution in how *information* is created, managed, analyzed, and applied. In addition, basic *information* will remain vitally *important*, as well as the *intelligence* we gather from data *ingestion* and preservation.

For fun, I put Love's lecture into ChatGPT to see how an AI program's summary would compare with mine (abridged version):

This article highlights the evolving role of medical librarianship and the necessity for librarians to adapt in an increasingly digital and research-driven world.

**Key Themes:**

1. Libraries Will Endure, But Must Adapt
2. Ownership vs. Access – A Critical Battle
3. Librarianship as a Research Discipline
4. Professional Development & Training
5. Quality Assurance in Information Services [21].

Not bad!

Continuing to demonstrate our *impact* will ensure our vitality and our professional *identity* among those with whom we partner and our employers. We need to never lose sight of the *implications* of assessing our *institutional* worth. We will *invest* time to *inventory* and develop *identified* skills, maintain our *integrity* and tenacity, and *immerse* ourselves within our *institutions* to provide quality health *information*. We will *interface* or *interact* with many *individuals* at their level of understanding and offer guidance in selecting accurate *information*. We will fight *illiteracy*, especially health *illiteracy*, and coach patients to comprehend their health care to make collective decisions with their providers.

I have emphasized the letter *I* throughout my lecture. I’d like to transition the many *I*s I see in this room (and virtually) to *we*. *We* collectively offer an *invaluable* service to others and to our *institutions*. *We* have transformed to adapt to the emerging trends of the day, new technologies, and new ways of developing and delivering our professional skills. I believe we can and will continue to transform and adapt, as we continue our professional journey.

Bunting summarized in her review of 30 years of past Janet Doe Lectures. “Overall, the opportunities, challenges, and changes described are welcome, presented in a positive light, and illustrate the adaptability of the profession” [22].

Quoting Matheson from her 1994 lecture when she referenced other Janet Doe lecturers, “All have written about what they hold nearest and dearest to their professional hearts, seeking to inform, to provide insight, to inspire, and even to entertain” [23]. I hope I have entertained you today.

We are the future, but only if we take care of *I* or *U* along the way. As Sollenberger recommended in her lecture – “compassion, kindness, thoughtfulness, caring and joy – these belong in our workplaces just as much, if not more, than searching skills, or strategic planning, or big-picture visions” [24].

My *instructions* for you are to be kind to each other, be kind to yourself, and be kind to mankind. An *interesting* and bright future awaits you. Embrace what is to come, maintain your *integrity*, your *initiative*, your *imagination* and your *intrigue*, and throw in some *innovative* fun along your *iterative* journey.

Thank you!
